# Investigation of Risk Of Bias due to Unreported and SelecTively included results in meta-analyses of nutrition research: the ROBUST study protocol

**DOI:** 10.12688/f1000research.20726.2

**Published:** 2020-02-07

**Authors:** Matthew J. Page, Lisa Bero, Cynthia M. Kroeger, Zhaoli Dai, Sally McDonald, Andrew Forbes, Joanne E. McKenzie

**Affiliations:** 1 School of Public Health and Preventative Medicine, Monash University, Melbourne, VIC, 3004, Australia; 2 Charles Perkins Centre, School of Pharmacy, Faculty of Medicine and Health, The University of Sydney, Camperdown, NSW, 2006, Australia

**Keywords:** Bias, Publication bias, Systematic reviews, Diet, Food and Nutrition

## Abstract

**Background: **Dietary guidelines should be informed by systematic reviews (SRs) of the available scientific evidence. However, if the SRs that underpin dietary guidelines are flawed in their design, conduct or reporting, the recommendations contained therein may be misleading or harmful. To date there has been little empirical investigation of bias due to selective inclusion of results, and bias due to missing results, in SRs of food/diet-outcome relationships.

**Objectives: **To explore in SRs with meta-analyses of the association between food/diet and health-related outcomes: (i) whether systematic reviewers selectively included study effect estimates in meta-analyses when multiple effect estimates were available; (ii) what impact selective inclusion of study effect estimates may have on meta-analytic effects, and; (iii) the risk of bias due to missing results (publication bias and selective non-reporting bias) in meta-analyses.

**Methods: **We will systematically search for SRs with meta-analysis of the association between food/diet and health-related outcomes in a generally healthy population, published between January 2018 and June 2019. We will randomly sort titles and abstracts and screen them until we identify 50 eligible SRs. The first reported meta-analysis of a binary or continuous outcome in each SR (the ‘index meta-analysis’) will be evaluated. We will extract from study reports all study effect estimates that were eligible for inclusion in the index meta-analyses (e.g. from multiple instruments and time points) and will quantify and test for evidence of selective inclusion of results. We will also assess the risk of bias due to missing results in the index meta-analyses using a new tool (ROB-ME).

**Ethics and dissemination: **Ethics approval is not required because information will only be extracted from published studies. Dissemination of the results will be through peer-reviewed publications and presentations at conferences. We will make all data collected from this study publicly available via the Open Science Framework.

## Introduction

Suboptimal diet is one of the leading contributors to mortality and morbidity globally ^1^. Dietary guidelines, which provide recommendations on types and amounts of foods to consume and dietary patterns to adopt, are developed with the aim of reducing non-communicable disease attributable to diet. Systematic reviews (SRs) of the available scientific evidence often underpin dietary guidelines ^2^. However, if the SRs are flawed in their design, conduct or reporting, the recommendations contained therein may be misleading or harmful.

Bias in the results of SRs can arise through various processes ^3^. Systematic reviewers often face a multiplicity of results for particular outcomes in the included studies (e.g. there may be results for weight loss at multiple time points, each of which are presented unadjusted and adjusted for prognostic factors, such as age and sex) ^4, 5^. When multiple results for an outcome are available in study reports, systematic reviewers’ choice about which result to include in a meta-analysis may be influenced by the P value, magnitude or direction of the result – this is known as ‘selective inclusion of results’ ^6^. For example, instead of including the result for weight loss that arose from the time point considered *a priori* to be the most clinically important, or which was adjusted for the most appropriate set of prognostic factors, systematic reviewers may select a result simply because it was the largest in magnitude, or had the smallest P value.

Bias in meta-analyses can arise not only when systematic reviewers selectively include results, but also when results of some eligible studies are unavailable for inclusion ^7^. There is extensive evidence that shows many studies are never published ^8^, and that those studies that are published tend to have larger effect estimates than unpublished studies ^9^. The term ‘publication bias’ has often been used to describe this problem. In addition, published studies often omit results for some of the outcomes that were measured ^10^, with reported results more likely to be statistically significant than non-reported results ^11^. The terms ‘selective outcome reporting’ and ‘outcome reporting bias’ have been used to describe this problem, but we prefer the term ‘selective non-reporting bias’ as it emphasises the *non-reporting* of study results. Regardless of whether an entire study report or a particular study result is unavailable selectively (e.g. because the P value, magnitude or direction of the results were considered less favourable by the investigators), the consequence is bias in a meta-analysis because available results differ systematically from missing results. The term ‘bias due to missing results’ has recently been coined to describe the bias in meta-analyses that arises from non-publication or non-reporting of study results ^12^.

There has been little empirical investigation of bias due to selective inclusion of results and bias due to missing results in SRs with meta-analyses of the association between food/diet and health-related outcomes. The only known investigation of selective inclusion of results focused on meta-analyses of randomized trials of interventions for arthritis or depressive or anxiety disorders ^13^. Also, previous assessments of reporting biases in nutrition research have been limited to an exploration of publication bias in meta-analyses of the association between diet and cardiovascular disease or mortality ^14^. There has been no formal investigation of selective non-reporting of results in studies included in meta-analyses of food/diet-outcome relationships.

Therefore, the aim of this research is to investigate various biases in SRs with meta-analyses of the association between food/diet and health-related outcomes. The objectives are to explore:

(i) whether systematic reviewers selectively included study effect estimates in meta-analyses when multiple effect estimates were available;
(ii) what impact selective inclusion of study effect estimates may have on meta-analytic effects, and;
(iii) the risk of bias due to missing results in meta-analyses.

## Methods

### Overview of the study

We will systematically search for SRs with meta-analysis of the association between food/diet and health-related outcomes in a generally healthy population, published between January 2018 and June 2019. We will randomly sort titles and abstracts and screen them until we identify 50 eligible SRs. The first reported meta-analysis of a binary or continuous outcome in each SR (which we refer to as the ‘index meta-analysis’) will be evaluated. We will extract from study reports all study effect estimates that were eligible for inclusion in the index meta-analyses (e.g. from multiple instruments and time points), and will calculate a statistic – the Potential Bias Index ^15^ – to quantify and test for evidence of selective inclusion of results. The risk of bias due to missing results (arising from publication bias and selective non-reporting bias) in the index meta-analyses will also be assessed using a new tool (the Risk Of Bias due to Missing Evidence (ROB-ME) tool ^12^).

### Eligibility criteria for SRs

We will seek a sample of SRs with meta-analysis meeting the following criteria:

- includes studies of people of any ages (i.e. infants, children, adolescents, adults or elderly people) and backgrounds in the generally healthy population, including pregnant and breastfeeding women and people with common diet-related risk factors such as being overweight or having high blood pressure;
- includes randomized trials or non-randomized studies evaluating the effects of at least one type of food (e.g. whole grains, fruit) or at least one food-defined dietary pattern (e.g. high intake of processed meat) on any binary (e.g. mortality) or continuous (e.g. weight) health-related outcome;
- published from 1 January 2018 to 30 June 2019;
- written in English;
- includes citations for all studies included in the SR; and
- presents the summary statistics or effect estimate and its precision (e.g. 95% confidence interval) for each included study, and the meta-analytic effect estimate and its precision, for at least one meta-analysis of a binary or continuous outcome.

We will adopt the definition of “systematic review” used in the 2019 edition of the *Cochrane Handbook for Systematic Reviews of Interventions*: “A systematic review attempts to synthesize all empirical evidence that fits pre-specified eligibility criteria in order to answer a specific research question. It uses explicit, systematic methods that are selected with a view to minimizing bias, thus providing more reliable findings from which conclusions can be drawn and decisions made” ^16^. We will use the criteria adopted by Page *et al*. to identify articles as SRs with meta-analysis, i.e. we will include articles with explicitly stated methods of study identification (e.g. a search strategy), explicitly stated methods of study selection (e.g. eligibility criteria and selection process), and a meta-analysis of study results ^17^. We will not exclude articles based on the level of detail about the methods provided (e.g. articles with a line-by-line Boolean search strategy or just a list of the key words used in the bibliographic databases will both be considered to meet the criteria for an SR).

We will exclude:

- SRs that did not include any meta-analysis of a binary or continuous outcome;
- meta-analyses or pooled analyses of studies conducted outside the context of a SR (e.g. when individual participant data are combined from a set of cohort studies outside the context of a systematic review, e.g. see Zhong *et al*. ^18^);
- SRs that focus only on nutrient-specific associations with outcomes (e.g. those examining the effects of single nutrients such as folic acid, salt).
- SRs including studies that were restricted to people with a health condition (e.g. type II diabetes), people who are obese, or frail elderly people who are at risk of malnutrition (by ‘frail’ we mean a person who, following a minor stress, experiences a large deterioration in function and does not return to baseline homeostasis ^19^); and
- SRs that were co-authored by a member of the investigator team, because the assessment of bias may be influenced by the investigators’ prior involvement in the SR.

### Search for SRs

We will identify eligible SRs by searching PubMed and Epistemonikos (a database of SRs and other syntheses of health research that have been identified via systematic searches of the Cochrane Database of Systematic Reviews, PubMed, EMBASE, CINAHL, PsycINFO, LILACS, DARE, Campbell Library, JBI Database and EPPI-Centre Evidence Library). In PubMed, we will use the search strategy for diet- or nutrition-related studies developed and validated by Durao *et al*. ^20^, combine this strategy with the search string, “meta-analysis[pt] OR meta-analy*[ti]” to identify meta-analyses, and restrict the search from 1 January 2018 to 30 June 2019 (search strategy as extended data ^21^). Various search filters designed to retrieve SRs exist, however they are designed to retrieve SRs with or without meta-analyses, and have high sensitivity given the lack of consensus on what constitutes a SR. We have decided to use a more targeted PubMed search strategy (that is, articles will need to have been classified by the PubMed indexers using the “Meta-Analysis” [Publication Type] MeSH term, or include the terms “meta-analysis”, “meta-analyses”, “meta-analytic” or other variants in the title) given we are including SRs only if they present a meta-analysis. In Epistemonikos, we will use the following search strategy: (title:((nutri* OR diet*)) OR abstract:((nutri* OR diet*))) and limit the search from 1 January 2018 to 30 June 2019. 

### Selection process

***Selection of SRs***. We will export titles and abstracts into Microsoft Excel, remove all duplicate records, and randomly sort the remaining records. In the piloting phase, four investigators (MJP, CMK, ZD and SM) will independently assess 50 abstracts against the inclusion criteria (rating each as ‘Eligible’, ‘Ineligible’, or ‘Unsure’), to ensure the criteria are applied consistently. Following piloting, two investigators (MJP and one of CMK, ZD or SM) will independently screen titles and abstracts of 450 records. The full text of records rated as ‘Eligible’ or ‘Unsure’ will then be retrieved and assessed independently against the inclusion criteria by two investigators (MJP and one of CMK, ZD or SM). We will repeat this process (screening batches of 500 records) until the target sample of 50 SRs is met. Any discrepancies in screening decisions at each stage will be resolved via discussion, or by consultation with another investigator (JM or LB) where necessary.

***Selection of index meta-analyses***. From each SR one investigator (MJP) will select one meta-analysis of a binary or continuous outcome for assessment, stratifying the selection so that the final sample includes 25 binary and 25 continuous outcome meta-analyses. The selected meta-analysis will be the first meta-analytic result that is presented in the SR, and henceforth is referred to as the ‘index meta-analysis’. The index meta-analysis may be selected from the abstract, summary of findings table, or results section of the SR, depending on where the meta-analytic result is first reported in the publication. We have opted for the first meta-analytic result reported in the review because the first meta-analysis is likely to be the most (or one of the most) important analysis in the review on which the conclusions of the review are based. We will include meta-analyses regardless of the outcome domain measured (e.g. all-cause mortality, diabetes incidence), meta-analytic effect measure (e.g. odds ratio, mean difference), meta-analytic model (e.g. fixed-effect, random-effects), approach to modelling (i.e. frequentist or Bayesian) and number and type of included studies (i.e. randomized trial or non-randomized study). We will select only standard pairwise meta-analyses of aggregate data, not dose-response meta-analyses, network meta-analyses or meta-analyses of individual participant data.

We will retrieve all reports of studies that were included in each index meta-analysis, as cited by the systematic reviewers. Study reports could include journal articles, conference abstracts, dissertations, trial results posted in trials registers, or any other reports (e.g. government reports). If more than one reference for a study was cited by the systematic reviewers (e.g. a study was reported in multiple journal articles, or in a journal article and a conference abstract), we will retrieve all references cited. If study reports are written in languages other than English, we will attempt to translate them using Google Translate; we will exclude reports if the translation is not interpretable. We will also retrieve reports of studies that were included in the review but excluded from the meta-analysis by the systematic reviewers, to explore whether any eligible outcome data may have been missed from these reports or potentially excluded because of the nature of the results (e.g. statistical non-significance). We also expect that in some systematic reviews, citations to ‘near-miss’ studies along with reasons for exclusion, will be provided. For these reviews, we will scan the reasons for exclusion, and where these reasons indicate there was no useable data, we will retrieve the study to confirm that no data were available for inclusion in the review.

### Data collection and management

We will collect data using a standardised form with detailed guidance created in REDCap (Research Electronic Data Capture), a secure, web-based software platform designed to support data capture for research studies ^22, 23^. Four investigators (MJP, CMK, ZD and SM) will initially pilot the data collection form on a random sample of five index meta-analyses and all of their included studies, to ensure consistency in the data collection. Any discrepancies will be discussed amongst all four investigators, and the form and guidance will be revised as necessary. Following piloting, two investigators (MJP and one of CMK, ZD or SM) will independently collect data from all remaining index meta-analyses and included studies. Any discrepancies between the data extracted will be resolved through discussion or adjudication by a third investigator (JM or LB) if necessary.

***Data items to describe the general characteristics of the SRs***. We will record the following characteristics of each of the included SRs:

- Journal it is published in;
- Year of publication;
- Country of corresponding author of the SR;
- Whether or not a registration record (e.g. PROSPERO) or protocol for the SR was mentioned in the paper;
- Source of funding of the SR, classified as: ‘non-profit’, ‘for-profit’, ‘mixed’, ‘no funding’, or ‘not reported’ (for funding sources classified as ‘for-profit’ or ‘mixed’ we will record the name of the for-profit funder and classify each as ‘food industry’ or ‘other industry’);
- Conflicts of interest of systematic reviewers as disclosed in the SR report, classified as: ‘conflict of interest present’ when at least one systematic reviewer reported a financial conflict of interest of any type, excluding current study funding or industry employment; ‘no conflict of interest’ if all systematic reviewers stated they had no conflicts; and ‘missing’ if there was no disclosure statement ^24^; and
- Affiliation of the corresponding author of the SR, classified as ‘industry’ or ‘non-industry’ or ‘mixed’.

***Data items to describe the general characteristics of the index meta-analyses***. We will record the following characteristics of each of the index meta-analyses:

- Number of studies included in the meta-analysis;
- Number of participants included in the meta-analysis;
- Type of population investigated (i.e. participants that were eligible for inclusion in the index meta-analysis, as specified by the systematic reviewers);
- Type of interventions or exposures investigated;
- Type of studies included in the meta-analysis (classified as randomized trial or non-randomized study or both; we will also classify the type of non-randomized study as specified by the systematic reviewers, e.g. ‘non-randomized trial’, ‘cohort study’, ‘case-control study’);
- Outcome domain (e.g. cancer mortality, weight);
- Outcome primacy label provided by the systematic reviewers (classified as ‘primary’, ‘secondary’ or ‘not labelled’);
- Effect measure (e.g. odds ratio or mean difference); and
- Meta-analysis model (fixed-effect, fixed-effects or random-effects).

***Data items to evaluate selective inclusion of results in index meta-analyses***. To explore whether systematic reviewers selectively included study results in meta-analyses when multiple study results were available (e.g. included data at one time point ahead of another because the former was statistically significant), we need to determine which results were eligible for inclusion in the meta-analyses. Therefore, from each SR and its corresponding SR protocol (if available), we will extract descriptions of any eligibility criteria to select effect estimates to include in the index meta-analysis. Eligibility criteria comprise lists of intervention groups, measurement instruments, time points and analyses that were eligible for inclusion (e.g. systematic reviewers state that results that were either unadjusted or adjusted for at least one prognostic factor would be included in meta-analyses). We will also extract any decision rules to select effect estimates to include in the index meta-analysis. Decision rules comprise strategies to either select one effect estimate or combine effect estimates when multiple were available (e.g. systematic reviewers state that if the effects of multiple levels of red meat intake were available, only the contrast between the highest and lowest intake would be included in the meta-analysis).

We will classify each eligibility criterion and decision rule as a strategy to handle results arising from:

- multiple measurement instruments;
- multiple definitions/diagnostic criteria for an event;
- multiple cut-points on a continuous outcome measure;
- multiple time points;
- multiple intervention groups;
- final and change from baseline values;
- multiple analysis samples (e.g. intention-to-treat, per-protocol and as-treated);
- unadjusted and covariate-adjusted analyses;
- period and paired analyses in crossover trials;
- multiple information sources (e.g. journal article and trials results register);
- overlapping samples of participants (e.g. men only and older adults only); or
- another source of multiplicity ^25^.

We will also collect from each SR information about the study data included in the index meta-analysis. Such information will include the summary statistics for each group, and the effect estimate, measure of precision (e.g. standard error or confidence interval), and direction of the effect estimate for each included study, as displayed on the forest plot or in a table/text. We will also record whether systematic reviewers declared that study outcome data:

(i) were obtained from the study investigators because the data were not reported in the study publication;
(ii) required some algebraic manipulation of statistics in order to include the data in the meta-analysis (e.g. calculating a standard deviation from a 95% confidence interval of the mean);
(iii) originated from a report written in a language other than English which the systematic reviewers had translated into English, or;
(iv) required a method of imputation (such as imputing a missing standard deviation).

We will collect from study reports outcome data that could potentially be included in the index meta-analysis, according to the eligibility criteria and decision rules specified in the SR protocol, and with how the outcome was specified in the SR. By ‘outcome data’ we mean summary statistics (e.g. number of events, sample sizes) or an effect estimate (e.g. odds ratio) and some measure of precision (e.g. standard error, 95% confidence interval), or both if available. If no SR protocol is available, we will assume that no eligibility criteria and decision rules were pre-specified, even if some were reported in the published SR (‘worst-case scenario’ assumption), and extract all study outcome data based on how the outcome was specified in the SR. For example, a meta-analysis with the outcome ‘reduction in cardiovascular disease risk (Framingham Risk Score)’, that had no SR protocol, will have all available results for this particular outcome extracted from each study (e.g. at all time points, unadjusted and covariate-adjusted analyses, regardless of whether decision rules for these measures/analyses were stated in the published SR); however, no results for any other outcomes (e.g. using an alternative cardiovascular disease risk outcome calculated with a different algorithm) will be extracted.

When studies of more than two groups are encountered and each comparison is eligible for inclusion in a meta-analysis, systematic reviewers need to use a method that avoids multiple counting of participants. Systematic reviewers may choose to:

(i) include data from only one of the experimental intervention/exposure groups and the control group;
(ii) combine the two experimental intervention/exposure groups (e.g. sum the number of events across both intervention/exposure groups for binary outcomes or calculate the mean values for both experimental intervention/exposure groups for continuous outcomes), and compare this to the control group, or;
(iii) include data from each experimental intervention/exposure group as separate comparisons in the meta-analysis by dividing the sample size of the control group by the number of comparisons.

If systematic reviewers pre-defined a method to deal with multi-arm studies, we will follow that method when extracting data. If systematic reviewers did not pre-define a method to deal with multi-arm studies, and:

(i) selected one of the experimental intervention/exposure groups to include in the meta-analysis, we will extract the data required to calculate effect estimates for two comparisons: (a) experimental intervention/exposure A versus control, and (b) experimental intervention/exposure B versus control;
(ii) combined the two experimental intervention/exposure groups, we will extract the data required to calculate effect estimates for three comparisons: (a) experimental intervention/exposure A versus control, (b) experimental intervention/exposure B versus control, and (c) combination of intervention/exposure A and B versus control;
(iii) included multiple comparisons in the meta-analysis by dividing the control group in half, we will extract the data required to calculate effect estimates for two comparisons: (a) experimental intervention A versus control, and (b) experimental intervention B versus control, where for both comparisons the control group sample size will be halved.

The three methods above will be used when dealing with three-arm studies. We will extend these methods when there are more than three arms in a study.

All study effect estimates included in mean difference meta-analyses must be in units of one particular scale, although estimates can comprise a mixture of final values and change from baseline values. In contrast, the measurement scale units of study effect estimates included in standardised mean difference (SMD) meta-analyses can vary, although it is recommended that all estimates are final values, or change from baseline values, not a mixture. Therefore, for mean difference meta-analyses we will extract only data for the particular scale included by the systematic reviewer, but extract final and change from baseline values when available. For SMD meta-analyses that included final values, we will extract final values only for any relevant measurement scale (and vice versa for SMD meta-analyses that included change from baseline values).

We will only extract study outcome data that were reported completely, defined as reporting sufficient data for inclusion in a meta-analysis (i.e. reporting of an effect estimate and a measure of precision, or summary statistics that enable calculation of these); we will not request unpublished data (e.g. missing number of events) from study authors.

### Assessment of risk of bias due to missing results in index meta-analyses

We will assess the risk of bias due to missing results in each index meta-analysis using the ROB-ME (“Risk Of Bias due to Missing Evidence”) tool, introduced in the 2019 edition of the *Cochrane Handbook for Systematic Reviews of Interventions* ^12^. The ROB-ME tool is the first structured approach for assessing reporting biases in meta-analyses that considers both publication bias and selective non-reporting bias. Users first consider whether results known or presumed to have been generated by study investigators are unavailable for any of the included studies (e.g. by cross-checking what was pre-specified with what was reported by study authors). They then consider whether a meta-analysis is likely to be biased because of the unavailable results in the studies identified. Finally, users consider whether qualitative signals (e.g. non-comprehensive search) and the pattern of observed results suggest that additional results are likely to be missing systematically from the meta-analysis. The tool includes signalling questions, which aim to elicit information relevant to an assessment of risk of bias. Responses to the signalling questions feed into algorithms developed to guide users of the tool to judgements about risk of bias. The possible risk-of-bias judgements are: (i) low risk of bias, (ii) some concerns, and (iii) high risk of bias. 

Four investigators (MJP, CMK, ZD and SM) will independently perform ROB-ME assessments on each of the index meta-analyses. We will assign the four assessors to pairs and ask them to reach consensus on their ROB-ME assessments. Any discrepancies between the responses to signalling questions and risk-of-bias judgements that cannot be resolved via discussion will be adjudicated by a third investigator (JM or LB).

### Data analysis

***Descriptive analysis***. We will calculate descriptive summary statistics of the general characteristics of SRs and index meta-analyses. For categorical variables, we will present frequencies and percentages. For continuous variables, we will present means (with standard deviations) and medians (with interquartile ranges). We will calculate the frequencies and percentages of SR protocols and SRs reporting the different types of eligibility criteria and decision rules to select study effect estimates. We will calculate the proportion of studies that had multiple results available for inclusion in the index meta-analyses, and quantify the number of study effect estimates that were eligible for inclusion in the index meta-analyses, and the number of eligible effect estimates per study.

***Analysis of selective inclusion of results***. We will follow the analyses used in a previous investigation of selective inclusion of results, as described by Page *et al*. ^13, 15^. We will calculate a statistic (called the ‘Potential Bias Index’ (PBI)) to quantify and test for evidence of selective inclusion. In brief, this index is based on ordering the effect estimates in each study based on their magnitude and direction of effect, and then determining the position within that order where the effect estimate included in the index meta-analysis sits. The PBI is the weighted average rank position of the selected effect estimates, where the weights are the inverse of the number of effect estimates available per study. This weighting system therefore attributes greater priority to the rank positions of effect estimates where there are a larger number of effect estimates to choose from. The expression for PBI is:

$$PBI=\frac{\sum_{i=1}^{k}\frac{n_{i}(X_{i}-1)}{n_{i}-1}}{\sum_{i=1}^{k}n_{i}}$$

where there are *k* studies, *n _i_* is the number of effect estimates in study *i*, and *X _i_* is the rank of the selected effect estimate in study *i*. Derivation of the PBI, and a worked example, are provided in Page *et al*. ^15^.

The PBI ranges from 0 to 1. For meta-analyses comparing an experimental intervention/exposure with no intervention or placebo control, the PBI will have the value 1 when the effect estimate that is most favourable to the experimental intervention/exposure is always selected for inclusion from each study. By “most favourable” we mean the effect estimate that suggested the most benefit or least harm of the intervention/exposure. Conversely, the PBI will have the value of 0 when the effect estimate that is least favourable to the experimental intervention/exposure is always selected. For meta-analyses comparing different levels or patterns of intake of the same food (e.g. wholegrain bread consumed 5 days per week versus wholegrain bread consumed once a week), we will determine from the text of the review whether the systematic reviewers hypothesised that the higher or lower category would have the most benefit or least harm, and rank study effect estimates based on their favourability to the category of consumption considered to be most beneficial/least harmful. If we cannot determine the hypothesis of the systematic reviewers, we will exclude the meta-analysis from the PBI analyses. For meta-analyses comparing different foods/diets (e.g. vegan versus vegetarian diet), we will determine from the text of the review which intervention/exposure the systematic reviewers were most interested in evaluating (which we will consider the experimental intervention/exposure), and rank the study effect estimates based on their favourability to the experimental intervention/exposure. We will also perform a sensitivity analysis excluding meta-analyses comparing different foods/diets to examine the impact on the PBI.

Several methods for selecting effect estimates are acceptable in terms of not introducing bias, including (i) randomly selecting effect estimates, (ii) selecting effect estimates based on some clinical or methodological rationale or (iii) selecting the median effect estimate ^25^. If systematic reviewers employed selection methods ii and iii across the studies, we expect that the distribution of the selected effect estimates would be consistent with what we would observe under purely random selection, so on average, the selected effect estimates would be at the middle rank position and the PBI would take the value of 0.5. A PBI of 0.5 therefore suggests that there is no selective inclusion of the most or least favourable effect estimates. We will run a statistical test based on the PBI that has been constructed to test whether the observed selection of effect estimates is consistent with randomness of selection ^15^. Confidence intervals (95%) for the PBI will be obtained by bootstrap resampling ^26^.

For meta-analyses of binary outcomes, we will express all study effect estimates in terms of odds ratios (ORs) to enable ranking of them on the same metric. For meta-analyses of continuous outcomes, we will express all study effect estimates in terms of SMDs to enable ranking of them on the same metric. In addition, we will standardise the direction of effects so that ORs below 1 or SMDs below 0 represent effects that are more favourable to the experimental intervention/exposure. We will exclude from all PBI analyses index meta-analyses that included no studies with multiplicity of effect estimates, given there is no potential for selective inclusion of results in such meta-analyses.

We will also investigate the impact of any potential selective inclusion of study effect estimates on the magnitude of the resulting meta-analytic ORs and SMDs. For each of the meta-analyses of binary outcomes, we will calculate all possible meta-analytic ORs from all combinations of available study effect estimates. When the number of possible combinations is prohibitively large to calculate all combinations (i.e. >30,000), we will generate a random sampling distribution of 5,000 meta-analytic ORs. Each meta-analytic OR will be created by randomly selecting (with equal probability) an effect estimate for inclusion from each study comparison, and meta-analysing the chosen effects. For each distribution of generated meta-analyses, we will calculate (i) the percentile rank of the index meta-analytic OR; (ii) the median of all possible meta-analytic ORs, which represents the median of a distribution where study effect estimates were not selectively included, and (iii) the difference between the index meta-analytic OR and the median meta-analytic OR. When the difference between the index and median meta-analytic OR is minimal, we will conclude that any potential selective inclusion had a limited impact on the meta-analytic effect (worked example as extended data ^21^). We will use non-parametric statistics to describe these differences. We will also synthesise these differences using a random-effects meta-analysis model, with the meta-analytic weights based on the variance of the index meta-analytic OR estimate and the between-study variability estimated using the restricted maximum likelihood estimator. The Hartung-Knapp-Sidik-Jonkman confidence interval method will be used to calculate uncertainty in the combined differences ^27, 28^. We will repeat the above analyses for each of the meta-analyses of continuous outcomes by calculating meta-analytic SMDs rather than ORs. We will also convert all SMDs to log ORs by multiplying the SMDs by π/√3 = 1.814 ^29^, and will re-run the analyses including all 50 meta-analyses of ORs. We will quantify statistical inconsistency using the I ^2^ statistic ^30^ along with a 95% confidence interval.

We will conduct a fixed-effect meta-analysis of the PBI obtained in the current study with that estimated in the previous study by Page *et al.* ^13^. We will synthesize estimates of the PBI using a fixed-effect meta-analysis model because the number of included studies (n=2) will be too small to adequately estimate the between-study variance.

We will conduct subgroup analyses to explore whether the availability of an SR protocol or registration record; the SR being funded by the food industry; and the SR having at least one author disclosing a financial conflict of interest of any type, modifies the PBI. The confidence limits and P value for the difference in PBI between subgroups will be constructed using bootstrap methods ^26^, because statistical theory does not currently exist for the distribution of the difference between two PBIs. Specifically, the steps will be to (1) draw a bootstrap sample of trials from each subgroup (e.g. separate samples from ‘SRs funded by the food industry’ and ‘SRs not funded by the food industry’), (2) calculate the PBI for each subgroup, and (3) calculate the difference in the PBIs across the subgroups. This process will be repeated 2000 times. The confidence limits for the difference in PBI between subgroups will be the 2.5 ^th^ and 97.5 ^th^ percentiles of the bootstrap distribution of differences. The P value will be identified through an iterative search for the confidence level of the differences that touches the null value, where the P value will be calculated as 1 – confidence interval level.

A similar approach will be used to examine whether the PBI is modified by the number of available effect estimates. The approach will only deviate at step 3 where a regression of PBI on the number of available effect estimates will be fitted. The regression coefficient for the estimate of linear association will be stored. The bootstrap distribution of these regression coefficients will be used to calculate the confidence limits and P value as described above.

We will undertake a series of sensitivity analyses to investigate whether the PBI is robust to certain assumptions. For SRs without protocols or registration records, we will not be able to determine whether the eligibility criteria and decision rules to select results in the methods section of the SR were developed prior to or while undertaking the SR. Therefore, in these SRs, our primary calculation of the PBI will be based on the set of study effect estimates that were compatible with the assumption of ‘no pre-specified eligibility criteria or decision rules’. However, we will perform a sensitivity analysis where study effect estimates that were compatible only with the eligibility criteria and decision rules in the methods sections of the SR are included, to examine if this affected the PBI.

In our primary analysis of meta-analyses of continuous outcomes, we will convert all study effect estimates to SMDs to allow us to calculate the PBI in circumstances where multiple effect estimates were available for the same outcome domain, but measured on different scales. However, there is not necessarily a one-to-one relationship between the rank positions of effect estimates based on the mean difference and SMD (because the SMD additionally depends on the pooled standard deviation). Therefore, in a sensitivity analysis we will calculate the PBI based on the rank positions of the mean difference for the subset of study effect estimates that were measured on the same scale as the effect estimate included in the index meta-analysis. This will allow us to assess more accurately whether systematic reviewers had selectively included study effect estimates based on the magnitude of the mean difference in raw measurement scale units.

We anticipate that in some study reports, only an effect estimate and its standard error or 95% confidence interval will be presented (that is, the number of events or means and standard deviations per group will not be available). In this circumstance, to include the result in a meta-analysis, algebraic manipulation will be required. Algebraic manipulation may be considered challenging by some systematic reviewers, so effect estimates requiring algebraic manipulation may not have been considered by reviewers in the set of effect estimates to potentially include in the meta-analysis. For the primary calculation of the PBI, we will exclude study effect estimates that required algebraic manipulation; however, we will undertake a sensitivity analysis to explore whether the PBI is modified when we include these study effect estimates.

Finally, in our primary analysis of investigating the impact of any potential selective inclusion of study effect estimates on meta-analytic effect estimates, we will use the random-effects meta-analysis model to pool effect estimates when calculating the distribution of possible meta-analytic effects. We will also perform a sensitivity analysis to explore whether our primary analysis is modified when the distribution of meta-analytic effect estimates are calculated using a fixed-effect model.

***Analysis of risk of bias due to missing results***. We will calculate the agreement in responses to signalling questions and risk-of-bias judgements for the ROB-ME tool for consensus assessments across the pairs of reviewers using the weighted Kappa statistic and percentage agreement (both metrics will be presented with 95% confidence intervals) ^31^. We will interpret Kappa values as poor (≤0.00), slight (0.01–0.20), fair (0.21–0.40), moderate (0.41–0.60), substantial (0.61–0.80), or almost perfect (0.81–1.00) ^32^.

After reaching consensus on ROB-ME judgements across the reviewer pairs, we will calculate the frequency and percentage of index meta-analyses rated at ‘low risk of bias’, ‘high risk of bias’ or ‘some concerns’. We will conduct subgroup analyses to explore whether the SR being funded by a for-profit company, and the SR having at least one author reporting a conflict of interest of any type, were associated with the index meta-analysis being rated at high risk of bias due to missing results.

### Software

We plan to use Stata version 15 software ^33^ to conduct all analyses.

### Sample size

The sample size of 50 SRs with meta-analysis was primarily selected for feasibility reasons given our available resources. This was informed by the time taken to search, screen, extract data and undertake the analysis in a previous similar study ^13^. This sample size will allow estimation of the PBI to within a margin of error of ±0.05, assuming each meta-analysis includes an average of 8.1 studies, with 2.2 effect estimates per study (as observed in Page *et al*. ^13^).

### Study status

We have run the searches, piloted the screening form and started screening titles and abstracts against the eligibility criteria.

### Dissemination of information

Dissemination of the results will be through peer-reviewed publications and presentations at conferences. We will make all data collected from this study publicly available via the Open Science Framework.

## Discussion

To our knowledge, this is the first study to investigate bias due to selective inclusion of results and bias due to missing results in SRs with meta-analyses of the association between food/diet and health-related outcomes. Our study will address several aspects of selective inclusion of results that have not yet been explored, including the extent to which it occurs in meta-analyses of binary outcomes and in meta-analyses of non-randomized studies, and whether the practice is associated with funding source and conflicts of interest of the systematic reviewers. Our study will also provide the first evaluation of the measurement properties of the recently developed ROB-ME tool, which should provide insight into any possible revisions that may need to be made to the tool.

Our study has several strengths. We have pre-specified methods to identify, select and collect data from eligible SRs and studies, and will declare any modifications to the protocol in the final report. We will use systematic methods to identify eligible SRs with meta-analyses, including use of explicit inclusion criteria, sensitive search strategies, duplicate selection and collection of data from SRs and studies, and standardised and pilot-tested data collection forms.

There are also some limitations of our planned methods. Most of the studies included in the index meta-analyses are unlikely to have been registered or have analysis plans available, which will make it challenging to detect selective non-reporting of results reliably. For example, in a cross-sectional analysis of 264 randomized trials of nutrition and dietetics interventions published in 2016, only 62 (24%) were registered prospectively ^34^; the proportion of non-randomized nutrition studies that are prospectively registered is likely to be far lower. In addition, our investigations of whether selective inclusion of results and risk of bias due to missing results is associated with conflicts of interest of systematic reviewers relies on systematic reviewers declaring such interests in the SR report; however, previous research suggests that the level of conflict of interest disclosure in nutrition research articles is low ^35, 36^.

## Conclusion

Meta-analyses of nutrition research underpin the recommendations of dietary guidelines. Therefore, it is essential that the findings of such meta-analyses are robust. Our study will examine previously underexplored sources of bias in meta-analyses of nutrition research. The findings may have implications for the design, conduct and reporting of future SRs with meta-analyses of the association between food/diet and health-related outcomes.

## Ethics

Ethics approval is not required because information will only be extracted from published studies.

## Data availability

### Underlying data

No data are associated with the article.

### Extended data

Open Science Framework: Investigation of Risk Of Bias due to Unreported and SelecTively included results in meta-analyses of nutrition research: the ROBUST study. https://doi.org/10.17605/OSF.IO/TCVJQ ^21^

This project contains the following extended data:

- ROBUST_protocol_appendix_1_20190920.pdf (PDF containing study search strategy)
- ROBUST_protocol_appendix_2_20191219.pdf (PDF containing worked example of the potential impact of selective inclusion of results on meta-analyses)

Data are available under the terms of the Creative Commons Zero “No rights reserved” data waiver (CC0 1.0 Public domain dedication).
