# Supplementary material for: Investigation of Risk Of Bias due to Unreported and SelecTively included results in meta-analyses of nutrition research: the ROBUST study protocol
Source: F1000Res. 2020 Feb 7;8:1760. Originally published 2019 Oct 16. [Version 2] doi: 10.12688/f1000research.20726.2 (PMC7025772; doi:10.12688/f1000research.20726.2)
Supplement: Peer review history

# Peer review: Reviewer response for version 2

**DOI:** 10.5256/f1000research.24643.r59686

**Published:** 2020-02-14

**Authors:** Robbie C.M. van Aert

**Affiliations:** 1 Department of Methodology and Statistics, Tilburg University, Tilburg, The Netherlands

I am satisfied with the revisions of the study protocol.

 One remaining minor comment is that the authors write on page 8:

- "When the difference between the index and median meta-analytic OR is minimal, we will conclude that any potential selective inclusion had a limited impact on the meta-analytic effect."

What is considered here as a "minimal difference"? I recommend the authors to define what they see as a minimal difference.

I confirm that I have read this submission and believe that I have an appropriate level of expertise to confirm that it is of an acceptable scientific standard.

# Peer review: Reviewer response for version 1

**DOI:** 10.5256/f1000research.22794.r55246

**Published:** 2019-10-30

**Authors:** Joshua Wallach

**Affiliations:** 1 Department of Environmental Health Sciences, Yale School of Public Health, New Haven, CT, USA

In this study protocol, Page et al. outline a project focused on exploring potential biases due to selective inclusion of results and missing results in systematic reviews of food/diet-outcome relationships. In particular, the authors have 3 main objectives:

1. To explore whether systematic reviews selectively include study effect estimates in meta-analyses when multiple effects are available.
2. To explore what impact selective inclusion of study effect estimates may have on meta-analytic effects. 
3. To explore the risk of bias due to missing results in meta-analyses. 

In the introduction of the protocol, the authors clearly justify the rationale for, and objectives of, the study. As the authors outline, if systematic reviews are flawed, the recommendations that could inform dietary guidelines could be misleading or harmful. Nutrition/dietary exposures are difficult to evaluate in observational studies, and numerous prior empirical evaluations have suggested concerns related to various biases. 

 The study design outlined by the authors is appropriate. Furthermore, most of the methods are clear and are sufficient to allow replication by others. A few minor suggestions:

- It would be helpful if the authors provided more information about their search for SRs. In particular, the authors note that they will use a PubMed to identify meta-analyses. Previous studies have outlined difficulties searching for meta-analyses. Is there a validated search strategy that the authors considered for systematic reviews/meta-analyses?

Other comments:

- In the introduction, the authors highlight that systematic reviews evaluating the association between food/diet and health-related outcomes could inform dietary guidelines. Have the authors considered limiting their sample to reviews that actually were used to inform guidelines? 
- When selecting one meta-analysis, why focus on the first identified analysis? Could the authors have considered the primary outcome? Or, if the goal is identify potentially selectively reported outcomes, only focusing on those with results highlighted in the abstract? 
- When looking at decision rules to select effect estimates: what if a meta-analysis includes multiple analyses based on different decision rules? It seems like this would make it difficult to evaluate selective inclusion? 
- I am a bit confused by this statement "If no SR protocol is available, we will assume that no eligibility criteria and decision rules were pre-specified, even if some were reported in the published SR ('worst-case scenario' assumption), and extract all study outcome data based on how the outcome was specified in the SR." Why not use the information in the published SR? 
- Would it be helpful if the authors discussed the potential bias index in the introduction? This is an important part of the study, but it is not discussed until the data analysis. 

I confirm that I have read this submission and believe that I have an appropriate level of expertise to confirm that it is of an acceptable scientific standard.

# Response

**Published:** 2020-02-05

**Authors:** Matthew Page

**Affiliations:** Monash University, Australia

It would be helpful if the authors provided more information about their search for SRs. In particular, the authors note that they will use a PubMed to identify meta-analyses. Previous studies have outlined difficulties searching for meta-analyses. Is there a validated search strategy that the authors considered for systematic reviews/meta-analyses?

**AUTHOR RESPONSE: We decided to search PubMed and Epistemonikos for meta-analyses of food/diet-outcome relationships. In PubMed, we have used the following terms to identify meta-analyses: “** **meta-analysis[pt]** OR meta-analy*[ti]” **. That is, articles will need to have been classified by the PubMed indexers using the "Meta-Analysis" [Publication Type] MeSH term, or include the terms “meta-analysis”, “meta-analyses”, “meta-analytic” or other variants in the title. Various search filters designed to retrieve systematic reviews exist (see** **https://sites.google.com/a/york.ac.uk/issg-search-filters-resource/filters-to-identify-systematic-reviews** **). However, these are designed to retrieve systematic reviews with or without meta-analyses, and typically have high sensitivity given the lack of consensus on what constitutes a systematic review. We decided to opt for a more targeted PubMed search strategy for articles classified by database indexers or the authors themselves as meta-analyses, given we are including systematic reviews only if they present a meta-analysis.** **We believe this search strategy will be sufficiently comprehensive given the consensus on what constitutes a meta-analysis. The other database we are searching, Epistemonikos, has been populated by conducting systematic searches for systematic reviews (with or without meta-analyses) indexed in 10 databases (listed at** **https://www.epistemonikos.org/en/about_us/methods#** **). We have provided this additional information under “Search for SRs”.**

In the introduction, the authors highlight that systematic reviews evaluating the association between food/diet and health-related outcomes could inform dietary guidelines. Have the authors considered limiting their sample to reviews that actually were used to inform guidelines?

**AUTHOR RESPONSE: We decided not to limit our study to systematic reviews that were used to inform dietary guidelines for two reasons. First, investigators have found that most dietary guidelines are not underpinned by systematic reviews. For example, Blake et al. found that of 32 food-based dietary guidelines published from 2010 to 2016, only 10 (31%) were informed by previously published systematic reviews (** **https://www.ncbi.nlm.nih.gov/pubmed/29425371** **). Therefore, relying on dietary guidelines to identify systematic reviews may result in a smaller than desired sample. Second, the systematic reviews used to inform dietary guidelines are not likely to be current because of the time-lag between development and publication of guidelines. By limiting our focus to recently published systematic reviews (Jan 2018 to Jul 2019), we will be able to comment on current issues with transparency and risk of bias in reviews.**

When selecting one meta-analysis, why focus on the first identified analysis? Could the authors have considered the primary outcome? Or, if the goal is identify potentially selectively reported outcomes, only focusing on those with results highlighted in the abstract?

**AUTHOR RESPONSE: We have decided not to focus on the primary outcome of the systematic review because many reviews do not specify a primary review outcome. For example, Bassani et al. found that of 480 systematic reviews in dentistry indexed in PubMed in 2017, only 151 (32%) specified a primary outcome (** **https://www.ncbi.nlm.nih.gov/pubmed/30716451** **). Also, in a sample of reviews not restricted by topic that were indexed in MEDLINE in Feb 2014, 136/288 (47%) specified a primary outcome (** **https://www.ncbi.nlm.nih.gov/pubmed/27218655** **). Therefore, assuming that many recent reviews will not specify a primary outcome, we need an alternative rule to select meta-analyses that can be applied across all reviews. We have opted for the first meta-analytic result reported in the review because the first meta-analysis is likely to be the most (or one of the most) important analysis in the review on which the conclusions of the review are based.**

When looking at decision rules to select effect estimates: what if a meta-analysis includes multiple analyses based on different decision rules? It seems like this would make it difficult to evaluate selective inclusion?

**AUTHOR RESPONSE: In our previous study investigating selective inclusion of results (** **https://www.ncbi.nlm.nih.gov/pubmed/27121706** **), we found that it was always clear which decision rules applied to which meta-analysis, because the rule was applicable only to a particular outcome (e.g. authors specified a hierarchy of measurement scales for pain, and a hierarchy of scales for anxiety), or authors made it clear that the rule applied to all meta-analyses (e.g. stated that for all meta-analyses of continuous outcomes, final values were preferred over change from baseline values). We assume that the same level of specificity will occur in the systematic reviews examined in current study.**

I am a bit confused by this statement "If no SR protocol is available, we will assume that no eligibility criteria and decision rules were pre-specified, even if some were reported in the published SR ('worst-case scenario' assumption), and extract all study outcome data based on how the outcome was specified in the SR." Why not use the information in the published SR?

**AUTHOR RESPONSE: In our primary analysis, we will ignore the eligibility criteria and decision rules to select results that were specified in the published SR, because there is a risk that such criteria and rules were created by systematic reviewers post-hoc. For example, systematic reviewers may have examined the study results for different scales measuring satiety, and crafted a post-hoc decision rule for scales that prioritises inclusion of the result with the largest effect estimate or smallest P value. However, as noted in paragraph eight of the section on “Analysis of selective inclusion of results”, “…we will perform a sensitivity analysis where study effect estimates that were compatible only with the eligibility criteria and decision rules in the methods sections of the SR are included, to examine if this affected the PBI”.**

Would it be helpful if the authors discussed the potential bias index in the introduction? This is an important part of the study, but it is not discussed until the data analysis.

**AUTHOR RESPONSE: We believe it is best to leave the description of the Potential Bias Index until the data analysis section, because of the extensive detail that is required to describe the index. Including information about the index in the background may detract from the rationale for the study and the objectives.**

# Peer review: Reviewer response for version 1

**DOI:** 10.5256/f1000research.22794.r55247

**Published:** 2019-10-21

**Authors:** Robbie C.M. van Aert

**Affiliations:** 1 Department of Methodology and Statistics, Tilburg University, Tilburg, The Netherlands

The reviewed manuscript is a study protocol for studying the risk of bias due to unreported and selectively included results in meta-analyses of nutrition research. I really appreciate the effort the authors put in this detailed study protocol. Below I list some major and minor comments to improve the protocol.

**Major comments:**

1. If there is no systematic review protocol available, there is assumed that there were no eligibility criteria or decision rules pre-specified. I believe this is way too restrictive, because meta-analysts will most of the time have explained what their inclusion and exclusion criteria were in the paper. This is also what is acknowledged in the proposed study protocol, but there is still decided to not use this information.
  Why are the systematic reviews without study protocol not included as a separate category? It would be interesting to see whether risk of bias is smallest in the systematic reviews with study protocols, larger in the ones with a clear description in the paper, and largest in the ones without a clear description in a study protocol or the paper.
2. There is planned to also include study reports that were eligible for inclusion in the index meta-analysis but excluded in the systematic review. Do the authors of the study protocol expect that a list is included in any systematic review with the study reports that were excluded or are they planning to redo the literature search? If the authors will be relying on a list of excluded study reports, how are they planning to deal with situations were such a list is unavailable?
3. The first meta-analysis is the selected meta-analysis that will be included in the study. Is it expected that the first meta-analysis is equivalent to any other meta-analysis in the systematic review? Do the authors expect that the first meta-analysis is representative for the other meta-analyses in the systematic reviews? I can imagine that the first meta-analysis is on the main effect under study of a systematic review and that the risk of bias is expected to be the largest for this meta-analysis.

 

**Minor comments:**

1. P.3: The authors may want to consider to change the order of the second and third paragraph of the introduction section. I believe that it is more logical to start with explaining that not all studies are usually accessible for being included in a meta-analysis and then explain that systematic reviewers themselves can also purposefully select which studies to include.
2. P.3: An eligibility criterion of a systematic review to be included is that it can be either a randomized trial or a non-randomized study. Please consider using stratification here such that 50% of the included systematic reviews are randomized trials and 50% non-randomized studies. I believe that this may yield relevant insights as randomized trials are usually seen as of higher quality.
3. P.8: I have a hard time understanding what the planned analysis is for studying the impact of any potential selective inclusion of study effect estimates on the magnitude of resulting meta-analytic ORs and SMDs (last paragraph, left column). Are the authors planning to compute odds ratios for each possible constellation of the 2x2 table of each individual study and then meta-analyzing these odds ratios? I do not see how this will yield insight into the effect of selective inclusion of study effect estimates on the magnitude of the meta-analytic estimate. Moreover, did the authors already write syntax/code for this? If yes, please add this to the study protocol as well.
4. P.8: Related to my previous minor comment, I also do not understand why converting of SMDs to log ORs is necessary. Why not conducting such an analysis based on SMDs?
5. P.8: A fixed-effect meta-analysis is planned to be used to combine the PBI that will be observed in the planned study with a PBI obtained in an earlier study. I doubt whether it is useful to meta-analyze these to PBI-values. Why not interpreting the PBI-value that will be obtained independently from the previously obtained PBI-value? It is, of course, good to relate this PBI-value to the previously obtained one, but I do not really see the need for meta-analyzing the two. Especially not since there are only two PBI-values that can be combined, so it is perfectly possible to interpret the two independently of each other.
  If the authors still decide to use a fixed-effect meta-analysis for combining the PBI values, please report the I ^2^ statistic together with its confidence interval. The computed I ^2^ statistic will be very uncertain in case of two studies and it is important to acknowledge this uncertainty.
6. P.8: The effect of several independent variables on PBI will be tested by means of bootstrap methods. Why are not more conventional methods used for testing these independent variables such as regression analysis?
7. P.9: The impact of any potential selective inclusion of study effect estimates on meta-analytic effect estimates will be studied using random-effects meta-analysis. Please also report what estimator will be used for estimating the between-study variance in the random-effects meta-analysis.

I confirm that I have read this submission and believe that I have an appropriate level of expertise to confirm that it is of an acceptable scientific standard, however I have significant reservations, as outlined above.

# Response

**Published:** 2020-02-05

**Authors:** Matthew Page

**Affiliations:** Monash University, Australia

Major comments:

If there is no systematic review protocol available, there is assumed that there were no eligibility criteria or decision rules pre-specified. I believe this is way too restrictive, because meta-analysts will most of the time have explained what their inclusion and exclusion criteria were in the paper. This is also what is acknowledged in the proposed study protocol, but there is still decided to not use this information. Why are the systematic reviews without study protocol not included as a separate category? It would be interesting to see whether risk of bias is smallest in the systematic reviews with study protocols, larger in the ones with a clear description in the paper, and largest in the ones without a clear description in a study protocol or the paper.

**AUTHOR RESPONSE: In our primary analysis, we will ignore the eligibility criteria and decision rules to select results that were specified in the published SR, because there is a risk that such criteria and rules were created by systematic reviewers post-hoc. For example, systematic reviewers may have examined the study results for different scales measuring satiety, and crafted a post-hoc decision rule for scales that prioritises inclusion of the result with the largest effect estimate or smallest P value. However, as noted in paragraph eight of the section on “Analysis of selective inclusion of results”, “…we will perform a sensitivity analysis where study effect estimates that were compatible only with the eligibility criteria and decision rules in the methods sections of the SR are included, to examine if this affected the PBI”. We also state in paragraph seven of this section that “We will conduct subgroup analyses to explore whether the availability of an SR protocol or registration record…modifies the PBI”. In other words, we will compare selective inclusion in SRs with a protocol/registration entry versus SRs without.**

There is planned to also include study reports that were eligible for inclusion in the index meta-analysis but excluded in the systematic review. Do the authors of the study protocol expect that a list is included in any systematic review with the study reports that were excluded or are they planning to redo the literature search? If the authors will be relying on a list of excluded study reports, how are they planning to deal with situations were such a list is unavailable?

**AUTHOR RESPONSE: We have clarified in paragraph two of the section, “Selection of index meta-analyses”, that “We will also retrieve reports of studies that were included in the review but excluded from the meta-analysis by the systematic reviewers…)”. We expect to be able to identify which of the studies included in the review were omitted from the meta-analysis given that all (or nearly all) systematic reviews cite every study included in the review. We have added the following text to this paragraph: “We also expect that in some systematic reviews, citations to ‘near-miss’ studies along with reasons for exclusion, will be provided. For these reviews, we will scan the reasons for exclusion, and where these reasons indicate there was no useable data, we will retrieve the study to confirm that no data were available for inclusion in the review.” We will not redo the literature search.**

The first meta-analysis is the selected meta-analysis that will be included in the study. Is it expected that the first meta-analysis is equivalent to any other meta-analysis in the systematic review? Do the authors expect that the first meta-analysis is representative for the other meta-analyses in the systematic reviews? I can imagine that the first meta-analysis is on the main effect under study of a systematic review and that the risk of bias is expected to be the largest for this meta-analysis.

**AUTHOR RESPONSE: We do not expect the first meta-analysis will necessarily be representative of all other meta-analyses in the systematic review. However, the first meta-analysis is likely to be the most (or one of the most) important analysis in the review on which the conclusions of the review are based. For this reason, we have chosen to focus our assessment on the first meta-analysis.**

Minor comments:

P.3: The authors may want to consider to change the order of the second and third paragraph of the introduction section. I believe that it is more logical to start with explaining that not all studies are usually accessible for being included in a meta-analysis and then explain that systematic reviewers themselves can also purposefully select which studies to include.

**AUTHOR RESPONSE: We would prefer to retain the order of the second and third paragraph, as we think it is more logical to start with explaining how meta-analyses may be biased because of actions of review authors (i.e. selective inclusion of results), followed by actions beyond review authors’ control (i.e. non-reporting by study authors).**

P.3: An eligibility criterion of a systematic review to be included is that it can be either a randomized trial or a non-randomized study. Please consider using stratification here such that 50% of the included systematic reviews are randomized trials and 50% non-randomized studies. I believe that this may yield relevant insights as randomized trials are usually seen as of higher quality.

**AUTHOR RESPONSE: As noted under “Selection of index meta-analyses”, we are already stratifying the sample so that it includes 25 meta-analyses of binary outcomes and 25 meta-analyses of continuous outcomes. The screening of titles and abstracts conducted thus far suggests there is a strong association between outcome type and type of included study in meta-analyses of nutrition research. That is, meta-analyses of continuous outcomes (such as weight) are based typically on data from randomized trials, and meta-analyses of binary outcomes (such as all-cause mortality) are based typically on data from non-randomized studies. Therefore, we believe our current stratification approach (by outcome type) will lead to a roughly equal sample of meta-analyses of randomized trials and meta-analyses of non-randomized studies.**

P.8: I have a hard time understanding what the planned analysis is for studying the impact of any potential selective inclusion of study effect estimates on the magnitude of resulting meta-analytic ORs and SMDs (last paragraph, left column). Are the authors planning to compute odds ratios for each possible constellation of the 2x2 table of each individual study and then meta-analyzing these odds ratios? I do not see how this will yield insight into the effect of selective inclusion of study effect estimates on the magnitude of the meta-analytic estimate. Moreover, did the authors already write syntax/code for this? If yes, please add this to the study protocol as well.

**AUTHOR RESPONSE: We plan to generate all possible meta-analytic effects that could be generated from the various combinations of study effect estimates available. For some studies included in an index meta-analysis, there will only have been one possible effect estimate available from the study report. For these studies, that sole effect estimate will be included in every iteration of the meta-analysis. For studies that have multiple effect estimates that are eligible for inclusion in the index meta-analysis (e.g. based on multiple scales or time points), we will randomly select a study effect estimate to include in each iteration of the meta-analysis. We will repeat this process until every possible meta-analytic effect estimate is computed (unless the number of possible combinations is prohibitively large to calculate all combinations (i.e. >30,000)). After calculating all possible meta-analytic effect estimates that the systematic reviewers could have generated, we will compare the meta-analytic effect estimate that was reported by the systematic reviewers with the median of all possible meta-analytic effect estimates that could have been generated. If the meta-analytic effect estimate presented by the systematic reviewers is much greater in magnitude than the median of all possible meta-analytic effect estimates, this suggests that systematic reviewers’ decision to selectively include study effect estimates had a considerable impact on the meta-analytic effect estimate reported. We have uploaded a worked example to the Open Science Framework (** **https://osf.io/pn38b/** **).**

P.8: Related to my previous minor comment, I also do not understand why converting of SMDs to log ORs is necessary. Why not conducting such an analysis based on SMDs?

**AUTHOR RESPONSE: To undertake the analysis including all 50 meta-analyses, we will have to use the same effect measure. Of the 50 included meta-analyses, 25 will be of continuous outcomes using the SMD, and 25 will be binary outcomes using the OR. We therefore need to either convert the SMDs to (log)ORs or the (log)ORs to SMDs. Given that we have an equal number of effect estimates to transform, we had no particular reason to adopt transforming (log)ORs to SMDs ahead of SMDs to (log)ORs.**

P.8: A fixed-effect meta-analysis is planned to be used to combine the PBI that will be observed in the planned study with a PBI obtained in an earlier study. I doubt whether it is useful to meta-analyze these two PBI-values. Why not interpreting the PBI-value that will be obtained independently from the previously obtained PBI-value? It is, of course, good to relate this PBI-value to the previously obtained one, but I do not really see the need for meta-analyzing the two. Especially not since there are only two PBI-values that can be combined, so it is perfectly possible to interpret the two independently of each other. If the authors still decide to use a fixed-effect meta-analysis for combining the PBI values, please report the I2 statistic together with its confidence interval. The computed I2 statistic will be very uncertain in case of two studies and it is important to acknowledge this uncertainty.

**AUTHOR RESPONSE: We believe there is value in meta-analysing the two PBI estimates. Doing so will yield a single estimate with greater precision. We plan to present a forest plot with estimates of PBI from the two studies, along with the combined PBI. Given there are only two estimates, we are not convinced that the I2 statistic will provide a useful quantification of inconsistency, and not more than can be obtained from visually inspecting the overlap in confidence intervals of the PBIs from each study.**

P.8: The effect of several independent variables on PBI will be tested by means of bootstrap methods. Why are not more conventional methods used for testing these independent variables such as regression analysis?

**AUTHOR RESPONSE: We plan to use bootstrap methods to estimate the confidence limits and P values since the PBI is a complex metric for which statistical theory concerning its distribution does not currently exist. We now provide more specific detail about our proposed bootstrap methods.**

P.9: The impact of any potential selective inclusion of study effect estimates on meta-analytic effect estimates will be studied using random-effects meta-analysis. Please also report what estimator will be used for estimating the between-study variance in the random-effects meta-analysis.

**AUTHOR RESPONSE: We stated in paragraph five of the section “Analysis of selective inclusion of results”, that the between-study variability will be “…estimated using the restricted maximum likelihood estimator”.**
